# An Autochthonous Outbreak of the SARS-CoV-2 P.1 Variant of Concern in Southern Italy, April 2021

**DOI:** 10.3390/tropicalmed6030151

**Published:** 2021-08-12

**Authors:** Daniela Loconsole, Anna Sallustio, Francesca Centrone, Daniele Casulli, Maurizio Mario Ferrara, Antonio Sanguedolce, Marisa Accogli, Maria Chironna

**Affiliations:** 1 Department of Biomedical Sciences and Human Oncology-Hygiene Section, University of Bari, 70124 Bari, Italy; daniela.loconsole@uniba.it (D.L.); francesca.centrone.fc@gmail.com (F.C.); accoisa@gmail.com (M.A.); 2 Hygiene Unit, University Hospital Consortium Policlinico, 70124 Bari, Italy; annasallustio@libero.it (A.S.); daniele.casulli@hotmail.com (D.C.); 3 Local Health Unit of Bari, Department of Prevention, 70132 Bari, Italy; mauriziomario.ferrara@asl.bari.it (M.M.F.); antonio.sanguedolce@asl.bari.it (A.S.)

**Keywords:** outbreak, P.1 variant, Gamma variant, SARS-CoV-2 infection, COVID-19, whole-genome sequencing

## Abstract

The SARS-CoV-2 P.1 variant of concern (VOC) was first identified in Brazil and is now spreading in European countries. It is characterized by the E484K mutation in the receptor-binding domain, which could contribute to the evasion from neutralizing antibodies. In Italy, this variant was first identified in January 2021. Here, we report an autochthonous outbreak of SARS-CoV-2 P.1 variant infections in southern Italy in subjects who had not travelled to endemic areas or outside the Apulia region. The outbreak involved seven subjects, three of whom had received a COVID-19 vaccine (one had received two doses and two had received one dose). Four patients had a mild clinical presentation. Laboratory investigations of nasopharyngeal swabs revealed that all strains were S-gene target failure-negative and molecular tests revealed they were the P.1 variant. Whole-genome sequencing confirmed that five subjects were infected with closely related strains classified as the P.1 lineage. The circulation of VOCs highlights the importance of strictly monitoring the spread of SARS-CoV-2 variants through genomic surveillance and of investigating local outbreaks. Furthermore, public health measures including social distancing, screening, and quarantine for travelers are key tools to slow down the viral transmission and to contain and mitigate the impact of VOC diffusion, and rapid scaling-up of vaccination is crucial to avoid a possible new epidemic wave.

## 1. Introduction

In December 2020, the European Center for Disease Control and Prevention (ECDC) first reported the spread of a new SARS-CoV-2 variant of concern (VOC) characterized by multiple spike protein mutations and mutations in other genomic regions, called VOC 202012/01—lineage B.1.1.7, in the UK, and labeled Alpha variant by the World Health Organization [1,2]. A few weeks later, a new ECDC risk assessment described the emergence of two new VOCs, namely, the 501Y.V2 variant (Beta variant), which was isolated in South Africa, and the P.1 variant (Gamma variant), which was identified in Brazil, mostly in the Amazonas state [3]. The overall risk associated with the introduction and community spread of these VOCs was assessed as being high/very high [3]. In May 2021, the SARS-CoV-2 B.1.617.2 Delta variant emerged in India and has spread all over the world [4]. At the time of writing, all these lineages seem to have almost replaced the previous circulating viruses in the geographic regions and the Delta VOC, in particular, shows very high probability of becoming the dominant circulating strain in the EU/EAA [4,5,6,7]. The spread of VOCs with a high transmission potential poses a serious risk in terms of virulence, potential reinfections, and antibody responses to and efficacies of vaccines [4,8]. 

The P.1 variant is characterized by 11 amino acid changes in the spike protein, three of which are located in the receptor-binding domain (RBD) [3]. These amino acid changes are L18F, T20N, P26S, D138Y, R190S, K417T, E484K, N501Y, H655Y, T1027I, and V1176F [3]. The 501Y.V2 and P.1 variants are both characterized by the E484K mutation in the RBD, which could contribute to the evasion from neutralizing antibodies [9,10]. Cases of reinfection caused by SARS-CoV-2 strains carrying the E484K mutation have been described [11]. The P.1 variant was first identified in Japan in four travelers from Brazil, but there was no indication it was associated with more severe disease [8]. However, recent studies reported evidence that disease severity is increased with this variant [12]. Moreover, an impact on transmissibility has also been demonstrated [13]. Retrospective analyses of samples collected in Manaus (Brazil) demonstrated the presence of the P.1 variant from November 2020, when case numbers of COVID-19 were high, and a rise of this variant from 0% to 87% in 7 weeks [13]. Moreover, a statistically significant association between P.1 infection and a lower Cycle threshold (Ct) value in real-time PCR, which is an indirect index of viral load in different specimens [14], was reported [13]. 

In Europe, infections of the P.1 variant, as well as the B.1.1.7, B.1.351, and B.1.617.2 VOCs, have been associated with a higher risk of hospitalization and intensive care unit admission [4,12]. In Italy, the P.1 variant was first reported in January 2021 in three patients returning from Brazil [15]. Monthly national flash surveys conducted in Italy to estimate the prevalence of VOCs from February 2021, reported that the estimated prevalence of the P.1 variant increased from 0% in February 2021 to 11.8% in June 2021 [16]. In the Apulia region, the estimated prevalence of the P.1 variant remained below 1% [16].

Here, we report an autochthonous outbreak of SARS-CoV-2 P.1 variant infections in southern Italy occurred in April 2021, when strict non-pharmaceutical interventions (NPI) were mandatory and travels outside the regions were forbidden.

## 2. Results

Of the seven patients involved in the outbreak, six were members of the same family and one was a friend of the index case. The demographic and clinical characteristics of the patients are shown in Table 1.

The index case (patient 1) was a healthy 45-year-old man who resided in a small town with about 13,000 inhabitants in the province of Bari, Italy, and presented with high-grade fever (>39 °C), sore throat, diarrhea, anosmia, and ageusia on 20 April 2021. He reported no history of travel in any area endemic for the SARS-CoV-2 P.1 variant or travel out of the Apulia region because travel was forbidden by national health authorities, nor contacts with any SARS-CoV-2 positive case. The patient tested positive for SARS-CoV-2 on 21 April 2021. His wife and other household members also tested positive for SARS-CoV-2 and were promptly quarantined. Contact tracing revealed that 30 subjects were contacts of the index case and his household members. All of them were tested for SARS-CoV-2 infection. Among these contacts, only a friend of the index case who showed symptoms on 26 April 2021, tested positive (patient 7). Epidemiological investigation revealed that the contact with the index case occurred on 20 April 2021 during football training. Patients 3, 5, and 7 had mild clinical presentation, with symptoms developing between 21 and 26 April 2021. Patient 3 was fully vaccinated with two doses of the BNT162b2 COVID-19 vaccine. The first dose was administered on 8 March 2021, and the second dose was administered on 30 March 2021, in accordance with the recommended schedule. Patients 4 had received one dose of the BNT162b2 COVID-19 vaccine on 19 April 2021, and patient 7 had received one dose of the ChAdOx1-S COVID-19 vaccine on 29 March 2021 (Table 1).

**Figure 1:**
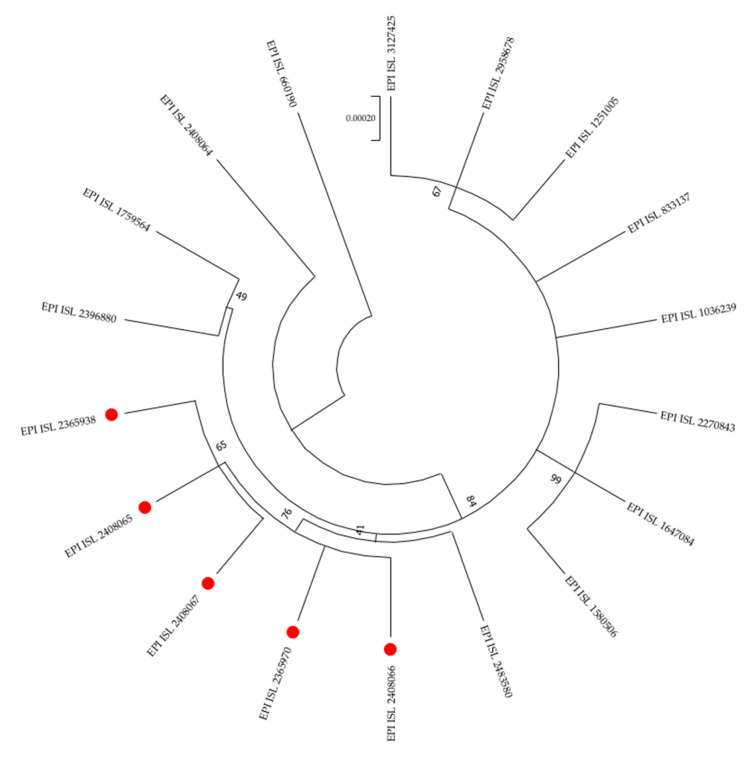
Phylogenetic tree of 18 SARS-CoV-2 full genome sequences, including the five genomes examined in this study (red dots). The reference SARS-CoV-2 genome (GISAID accession number: EPI_ISL_660190) was included to root the tree. P.1 reference strain has been used to construct the tree (GISAID accession numbers: EPI_ISL_833137). Other Italian P.1 SARS-CoV-2 sequences have been used for comparison (GISAID accession numbers: EPI_ISL_2408064, EPI_ISL_1759564, EPI_ISL_2396880, EPI_ISL_2483580, EPI_ISL_1580506, EPI_ISL_1647084, EPI_ISL_2270843, EPI_ISL_1036239, EPI_ISL_1251005, EPI_ISL_2958678, EPI_ISL_3127425). Evolutionary analyses were conducted in MEGAX by using the maximum likelihood method and Tamura-Nei model. The robustness of branching pattern was tested by 1000 bootstrap replications. Bootstrap values are reported. The scale bar indicates nucleotides substitutions per site.

The seven patients involved in the outbreak tested positive for SARS-CoV-2 by real-time PCR and all were S-gene target failure (SGTF)-negative. Therefore, samples were subjected to molecular screening for variants and designated the P.1 variant because of the presence of the K417T, E484K, and N501Y spike mutations. Whole-genome sequencing was performed of all seven strains, but high-quality SARS-CoV-2 genome sequences were only obtained for patients 1, 3, 4, 5, and 7. These were imputed into the PANGOLIN tool for lineage classification [17] and classified as the P.1 lineage. The sequences were deposited in the GISAID database (www.gisaid.com, accessed on 11 August 2021). The accession numbers are reported in Figure 1. The phylogenetic tree showed closely related strains, thus suggesting a common source of exposure (Figure 1).

## 3. Discussion

Virus importation associated with travel has been the key driver of viral spread since the first wave of the COVID-19 pandemic in Italy [18]. Cases of imported SARS-CoV-2 variant P.1 infection were first reported on 7 January 2021 in Central Italy in travelers returning from Brazil, and this variant was also subsequently identified in northern and southern Italy [15]. All direct flights from Brazil to Italy were cancelled on 16 January 2021. Another case of P.1 infection was identified in a traveler returning from Brazil on 17 January 2021, thus confirming the risk of introducing variants via indirect flights [19]. An in-depth molecular epidemiological analysis performed by Di Giallonardo et al. showed intensive local transmission of the P.1 variant in Italy after its travel-linked introduction [20]. In the Apulia region, the P.1 variant was first identified through a flash national survey on 20 April 2021 [21]. It was identified in a patient without risk factors for P.1 variant infection (i.e., travel or contact with a P.1 variant-positive case). Thereafter, we documented the circulation of the P.1 variant in two more provinces of the Apulia region (data not published). The present study reports an outbreak of the SARS-CoV-2 P.1 VOC in southern Italy. Among cases, no subjects with known travel history, nor contacts with other SARS-CoV-2 positive subjects were identified. Therefore, despite lockdown restrictions being imposed in Italy from December 2020, the cluster described here is concerning and suggests a local ongoing transmission, although at a low level, of this variant in the Apulia region. 

The symptomatic cases of the outbreak here described showed a mild clinical presentation. Recently, de Siqueira et al. also reported a familial cluster of five cases, with three having severe disease, one of whom died [22]. We could speculate that the difference in clinical presentation between this previous study and the cases described here could be related to the fact that three of our seven cases had received at least one dose of a COVID-19 vaccine, which may have protected them against severe disease. Of note, patient 4 received the first vaccine dose only a few days before being diagnosed and was asymptomatic, while patient 3 was fully vaccinated and received the second dose 1 month before the clinical onset of symptoms. As previously reported for the B.1.1.7 lineage VOC in the Apulia region [23], the P.1 variant raises concerns about such strains causing possible symptomatic post-vaccination infections. Some studies demonstrated that samples of vaccinated and convalescent people exhibit lower neutralization activity against SARS-CoV-2 strains harboring the E484K spike mutation [24], showing the need to induce the highest neutralization titers through vaccination. According to the European approach, the Italian national guidelines indicate that the booster vaccination should be postponed to provide more subjects with a first vaccination [25]. However, this approach will result in a lower level of neutralizing antibodies and could leave some vaccinees unprotected in the context of the rising spread of SARS-CoV-2 variants [24,26]. 

This study has some limitations. First, only five in seven samples were successfully subjected to WGS. However, the molecular screening for variants and the epidemiological linkage suggested the presence of SARS-CoV-2 P.1 lineage in all cases. Second, serum samples after vaccination and before symptom onset for anti-spike IgG detection were not available. Nevertheless, due to the incomplete vaccination schedule for two subjects, we could hypothesize a low humoral response to vaccination.

In Italy, where the vaccination campaign is accelerating but has not yet reached sufficient coverage, the spread of variants with higher transmissibility may have a significant impact, also in the light of the reduction of NPI. The current scenario characterized by the circulation of multiple VOCs and, in particular, of the Delta VOC [4], highlights the importance of closely monitoring the spread of SARS-CoV-2 variants through genomic surveillance and of investigating local outbreaks. Furthermore, maintaining public health measures including social distancing, screening, and quarantine for travelers are key tools to slow down the viral transmission and to contain and mitigate the impact of VOCs diffusion on the National Health Service. Finally, rapid up scaling of vaccination in Italy is crucial to avoid a possible new epidemic wave, particularly in younger people who have not yet been vaccinated.

## 4. Materials and Methods

Seven patients were involved in the outbreak. Their clinical presentations were classified according to the National Institute of Health (NIH) clinical staging of COVID-19 disease [27]. Nasopharyngeal swabs were collected from all patients at the Local Health Unit of Bari (Italy) and were processed at the Laboratory of Molecular Epidemiology and Public Health of the Hygiene Unit (A.O.U.C. Policlinico Bari), which is the coordinator of the Regional Laboratory Network for SARS-CoV-2 diagnosis in the Apulia region. RNA was extracted using a MagMAX Viral/Pathogen Nucleic Acid Isolation Kit (Thermo Fisher Scientific, Waltham, MA, USA). The molecular test was performed using a three-target commercial multiplex real-time PCR assay targeting the N, ORF1ab, and S genes (TaqPath RT-PCR COVID-19 Assay; Thermo Fisher Scientific). SGTF was assessed to rule out the B.1.1.7 lineage VOC because SGTF can be considered a robust proxy of VOC 202012/01 [26,28]. SGTF-negative samples were screened for the presence of notable types of spike protein mutations (HV 69-70 deletion, N501Y, K417N, E484K, and K417T) using a commercial multiplex real-time PCR kit (Seegene Allplex SARS-CoV-2 Variants I Assay, Arrows Diagnostics, Genova, Italy). Whole-genome sequencing was performed using the Ion Torrent platform (Thermo Fisher Scientific, Monza, Italy). The library was prepared using an Ion AmpliSeq Library Kit Plus according to the manufacturer’s instructions and the Ion AmpliSeq SARS-CoV-2 RNA custom primer panel (Thermo Fisher Scientific, Monza, Italy). Quality control of AmpliSeq reads and their alignment to the complete genome of the SARS-CoV-2 Wuhan-Hu-1 isolate were performed using the Torrent Server of the Ion Torrent S5 sequencer with default settings. The aligned reads were utilized for both reference-guided assembly and variant calling. The quality metrics for the reference-based assemblies are as follows: 784,477 sequence reads (average length 149 bp), 674,160 mapped reads, GC% 40, and an average base coverage depth of 3222. The total genome size was 29,780 bp. Assembly was performed using the Iterative Refinement Meta-Assembler (IRMA) v.1.3.0.2, which produced a consensus sequence for the sample using a cut-off of >50% for calling single nucleotide polymorphisms. The whole-genome sequences have been deposited in the GISAID database (https://www.gisaid.com, accessed on 11 August 2021). Phylogenetic analysis was performed using MEGAX software.

## Figures and Tables

**Table 1:** Demographic and clinical characteristics of seven cases of SARS-CoV-2 P.1 variant infections.

Patient Number	Relationship with the Index Case	Age (Years)	Sex	Comorbidities	Date of Onset of Symptoms	Date of Diagnosis	Clinical Presentation	Vaccinated
1	Index case	45	Male	No	20 April 2021	21 April 2021	Mild	No
2	Wife	31	Female	No	-	29 April 2021	Asymptomatic	No
3	Father-in-law	73	Male	Diabetes, Hypertension	28 April 2021	28 April 2021	Mild	Yes, BNT162b2 (second dose on 30 March 2021)
4	Mother-in-law	69	Female	No	-	28 April 2021	Asymptomatic	Yes, BNT162b2 (first dose on 19 April 2021)
5	Brother-in-law	53	Male	No	21 April 2021	22 April 2021	Mild	No
6	Nephew	16	Male	No	-	12 May 2021	Asymptomatic	No
7	Friend	37	Male	No	26 April 2021	27 April 2021	Mild	Yes, ChAdOx1-S (first dose on 29 March 2021)

## Data Availability

All data regarding the patients and laboratory tests are available from the corresponding author by e-mail request.
